# Clinical Presentation and Risk Factors of Infective Endocarditis in the Elderly: A Systematic Review

**DOI:** 10.3390/jpm13020296

**Published:** 2023-02-07

**Authors:** Camelia Melania Budea, Felix Bratosin, Iulia Bogdan, Adrian Vasile Bota, Mirela Turaiche, Livius Tirnea, Carmen Nicoleta Stoica, Andrei Nicolae Csep, Bogdan Feciche, Silvius Alexandru Pescariu, Malina Popa, Adelina Mavrea, Bogdan Andrei Bumbu, Satya Sai Sri Bandi, Iosif Marincu

**Affiliations:** 1 Department of Ear-Nose-Throat, “Victor Babes” University of Medicine and Pharmacy Timisoara, Eftimie Murgu Square 2, 300041 Timisoara, Romania; fizedean.camelia@umft.ro; 2 Methodological and Infectious Diseases Research Center, Department of Infectious Diseases, “Victor Babes” University of Medicine and Pharmacy Timisoara, Eftimie Murgu Square 2, 300041 Timisoara, Romania; felix.bratosin7@gmail.com (F.B.); iulia-georgiana.bogdan@umft.ro (I.B.); bota.adrian1@yahoo.com (A.V.B.); mirela.turaiche@gmail.com (M.T.); liviustirnea@yahoo.com (L.T.); stoica.carmennicoleta@yahoo.com (C.N.S.); csep.andrei@gmail.com (A.N.C.); imarincu@umft.ro (I.M.); 3 Department of Urology, Satu-Mare County Emergency Hospital, Strada Ravensburg 2, 440192 Satu-Mare, Romania; dr.feciche@yahoo.com; 4 Department VI, Cardiology, “Victor Babes” University of Medicine and Pharmacy Timisoara, Eftimie Murgu Square 2, 300041 Timisoara, Romania; pescariu.alexandru@umft.ro; 5 Department of Pedodontics, Faculty of Dental Medicine, “Victor Babes” University of Medicine and Pharmacy Timisoara, Revolutiei Boulevard 9, 300041 Timisoara, Romania; 6 Pediatric Dentistry Research Center, Faculty of Dental Medicine, “Victor Babes” University of Medicine and Pharmacy Timisoara, Revolutiei Boulevard 9, 300041 Timisoara, Romania; 7 Department of Internal Medicine I, Cardiology Clinic, “Victor Babes” University of Medicine and Pharmacy Timisoara, Eftimie Murgu Square 2, 300041 Timisoara, Romania; mavrea.adelina@umft.ro; 8 Department of Dental Medicine, Faculty of Medicine and Pharmacy, University of Oradea, 410073 Oradea, Romania; 9 Malla Reddy Institute of Medical Sciences, Suraram Main Road 138, Hyderabad 500055, India; sssbandi09@gmail.com

**Keywords:** infective endocarditis, pathogens, mortality, risk factors, elderly patients

## Abstract

Infective endocarditis (IE) is a bacterial infection with high morbidity and mortality rates, particularly among the elderly. This systematic review was conducted to determine the clinical characteristics of IE in older adults and the risk factors that could lead to adverse outcomes. The research employed three databases (PubMed, Wiley, and Web of Science), with a primary search for studies that have described cases of IE in patients older than 65 years. From a total of 555 articles, 10 were selected for inclusion in the current study, comprising a total of 2222 patients with a confirmed diagnosis of IE. The primary findings were an increased incidence of staphylococcal and streptococcal infections (33.4% and32.0%, respectively), an increased prevalence of comorbidities, namely, cardiovascular disease, diabetes, and cancer, and a significantly greater mortality risk than the younger group. Regarding mortality risks, the pooled odds ratio for cardiac disorders was OR = 3.81, septic shock OR = 8.22, renal complications OR = 3.75, and advancing age OR = 3.54 were most frequently mentioned. Taking into consideration the fact that the majority of the elderly population suffers from significant complications and is unable to undergo surgery due to an increased risk of complications after the procedure, it is imperative that effective treatment methods should be investigated.

## 1. Introduction

Infective endocarditis (IE) is an infection of the heart’s inner lining, known as the endocardium [1] with a high mortality and morbidity rate [2,3]. It is estimated that the number of incident cases has increased by 128% from 1990 to 2019, with an increased age–specific incidence rate for the population over 60 years old, with an overall five-fold increased risk of IE compared with the general population of adults which stands between 3 and 7 cases per 100 thousand patients [4]. Considering the continuously rising incidence and moving towards the aging global population [4], studies need to focus on this specific cohort, considering that older individuals have a higher prevalence of comorbidities and present with nonspecific symptoms that make the diagnosis difficult [2]. Infective endocarditis is defined by a wide variety of nonspecific symptoms [3,5,6], with fever, chills, nausea, dyspnea, anorexia, weight loss, weakness and fatigue, headaches and shortness of breath [3,7]. 

To diagnose IE, blood cultures and cardiac imaging techniques are utilized, with echocardiography being the quickest and most utilized in elderly patients [2,3]. Types of echocardiography that have been used the most frequently are transesophageal echocardiography (TEE) and transthoracic echocardiography (TTE) [3]. In addition, it is advised that the Duke criteria should be employed in the diagnosis process whenever there is a suspicion of IE [1,2,8]. The Duke criteria consist of major and minor criteria, such as blood cultures, the use of echocardiography as methods of diagnosis, and, respectively, minor criteria that include symptoms, comorbidities, or other relevant past conditions [6]. A problem arising is that the clinical presentation of IE in the elderly makes the Duke criteria sensitivity lower, therefore potentially missing the diagnosis and delaying the life-saving treatment [2]. 

Despite advances in diagnostic and therapeutic approaches, there has been no reduction in the disease’s prevalence or severity, particularly among the elderly [3,9]. This rise may be attributed to the general aging of the population, greater survival of patients with congenital and valvular heart disease, and the increased use of catheters and other prosthetic devices, which has resulted in a higher prevalence of nosocomial endocarditis [6]. Nevertheless, the elderly experience different complications, either from comorbidities or their treatment, that could interact with the treatment of the IE process, or from severe complications following past surgeries [3,5]. Some complications that have been found to occur are heart failure, renal failure, delirium, and functional decline [3,6]. When it comes to the involved pathogens individuals of all ages are susceptible to contracting an infection caused by streptococci or staphylococci, although it appears that streptococci and enterococci become the causal pathogens as age increases [6], most commonly found in the elderly population being coagulase-negative staphylococci, *S. bovis* and enterococci [2,3]. 

The most prevalent risk factors for IE found among the elderly people are the increased number of comorbidities, the increased number of predisposing events, such as surgery or hemodialysis, the use of prosthetic materials, and dental procedures, and the increased rates of nosocomial infections that overall increase the odds of developing IE [3,8,10]. The repercussions of nosocomial infections present another challenge, resulting in a higher incidence of methicillin-resistant organisms among people of this particular age group [2]. Although studies state that surgery can minimize the mortality rate [11,12], when it comes to the older age group, the risks to which the elderly are exposed during surgery can keep the mortality rate at a high level [13,14], especially for the population above 80 years old [15]. Complications that may occur following the surgical treatment for the elderly are usually sepsis, kidney failure, pneumonia, and postoperative delirium [2,13]. Nevertheless, early surgical intervention remains associated with lower in-hospital mortality [12]. Surgery is still advised, despite the risks involved, because it is currently the only method that can successfully replace the involved valves and eliminate the infection. [11]. 

Although IE was primarily classified as acute, subacute, or chronic [1,5], there has been a shift in its classification, which is now based on multiple factors, such as the source of acquisition or the individual’s background, such as intravenous drug users or the elderly [7]. Another distinction is also being made between community-acquired IE and healthcare-associated IE, such as nosocomial IE, which has been more prevalent in the past years for the aging population, along with comorbidities and staphylococcal infections [16]. When it comes to individual background, it seems that IE has increased negative outcomes for the elderly and for the younger population that are intravenous drug users [8,11].

There have been a lot of reviews that have focused on the prevalence and etiology of infective endocarditis [8,10,17], but there have only been a few that have focused on the specific age group that is more vulnerable, which comprises elderly individuals over the age of 65 years [3]. Infective endocarditis has a poor prognosis for this particular population [5,18]. Therefore, the purpose of this systematic review was to have a better understanding of the clinical presentation of endocarditis and to investigate its risk factors related to mortality in the elderly individuals.

## 2. Materials and Methods

### 2.1. Review Protocol

This systematic review was conducted in December 2022, including research published until December 2022, utilizing three electronic databases: PubMed, Web of Science, and Wiley. The following key terms were included when carrying out this research: “infective endocarditis” AND “risk factors” OR determinants AND senior OR old OR “older age” OR geriatric OR elder OR elderly AND mortality OR “mortality rate” OR outcomes OR “clinical outcomes” OR “clinical consequences.” To limit the results, disease and age-related terms were required to be stated in the abstract. In addition, the search was restricted to journal articles written in English.

All relevant scientific papers studying the clinical presentation of infective endocarditis and its risk factors in the elderly were included in the analysis by using a structured and methodical search approach, which was carried out in accordance with the Preferred Reporting Items for Systematic reviews and Meta-Analyses (PRISMA) criteria and the International Prospective Register of Systematic Reviews (PROSPERO) guidelines. The current systematic review was registered to the Open-Science Framework (OSF) platform.

This systematic review aimed to answer the following questions:

- What are the most common clinical features and pathogens involved in infective endocarditis among elderly patients?
- What are the most commonly involved risk factors for mortality among elderly patients?

### 2.2. Data Extraction

All information was gathered from the articles’ text, tables, figures, and online supplemental resources. The selection procedure comprised the elimination of duplicate entries, abstract screening, and full-text screening. Reference lists from the retrieved articles were manually examined for relevant information. Inclusion criteria comprised: (1) studies discussing the topic of infective endocarditis, (2) studies that included patients older than 60 years, and (3) studies presenting original observations regarding the clinical presentation and risk factors for infective endocarditis. The following variables were considered to be reported in the current review: country of the study, the cohort size, patients’ age, gender, causative pathogens, comorbidities, type of infection (community-acquired, healthcare-associated), mortality rate, and mortality risk factors. 

**Figure 1:**
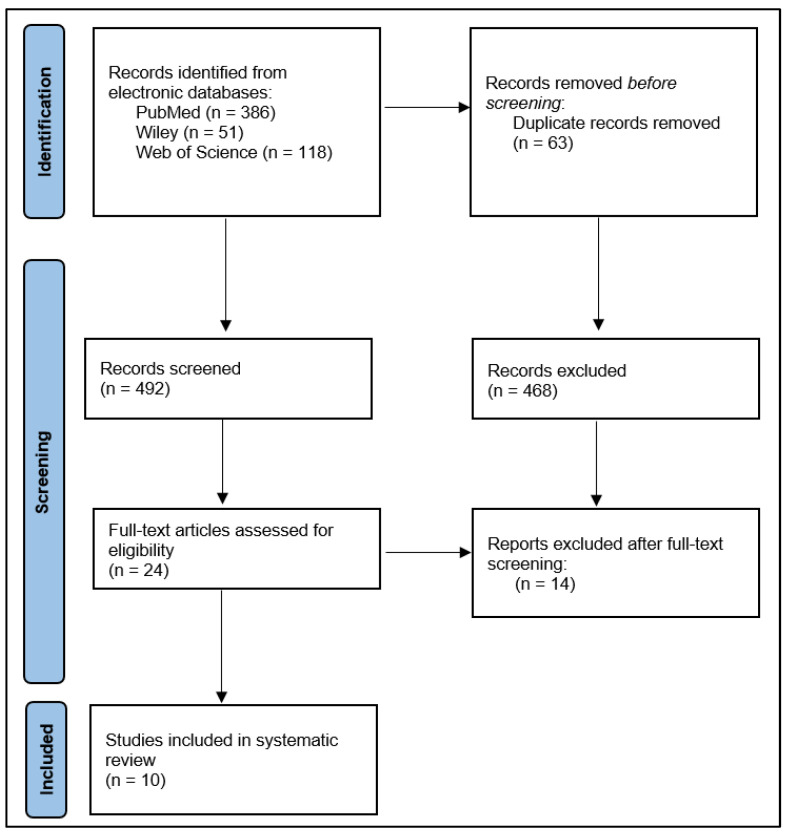
PRISMA Flow Diagram.

The initial results of the search revealed 555 articles, 63 of which were duplicates. After excluding 468 papers based on their abstracts, we analyzed 24 full-text articles, and from those, we chose 10 to include in our systematic review [19,20,21,22,23,24,25,26,27,28]. Exclusions were made for these primary reasons: (1) the average age of the study population was under sixty years old; (2) a lack of data reporting risk variables; (3) case reports, case series, reviews, and short communications were also excluded. The remaining data were evaluated by two different investigators independently, and complete texts were obtained only if both decided that the paper should be included. Pooled odds ratios of the reported risk factors were calculated using the meta-analysis function of the MedCalc v.26 statistical software. Figure 1 shows the stages that were followed in performing this systematic review search.

## 3. Results

### 3.1. Studies’ Characteristics 

All included studies were observational; seven were retrospective studies [19,21,23,24,25,28], while the other three [22,26,27] were prospective studies. The majority of the research was conducted in Europe, specifically in Romania [19], France [20,21], and Portugal [25]. This was followed by two studies conducted in the United States of America [22,23] and one study conducted in Japan [24]. Articles included had a wide range of publication years, from 1998 to 2022. Two studies only reported the clustered age, namely, above 65 years [22] and above 64 years [26], respectively. For the rest of the studies, the mean age was approximately 70. Except for one study that did not report the gender prevalence [23], every other study reported a higher prevalence of males. Demographic data can be found in Table 1. 

### 3.2. Clinical Outcomes and Risk Factors

**Figure 2:**
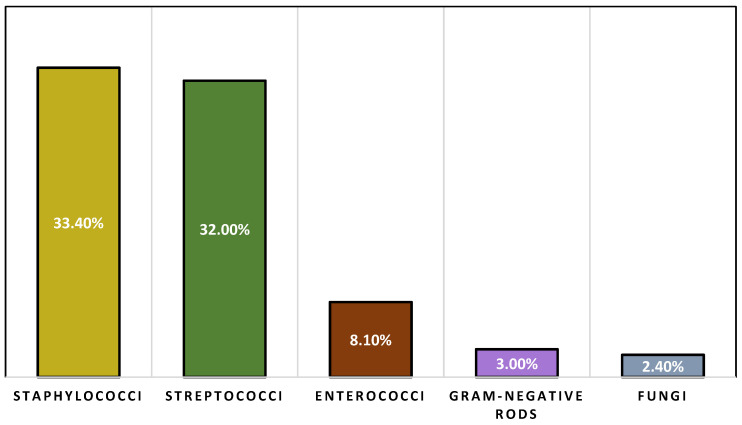
Prevalence of pathogens across studies.

Regarding the nature of the infection, half of the studies had a greater incidence of community-acquired IE [20,24,25,26,28], two of the studies reported more cases of health-care-associated IE [19,22], and three studies did not describe the source of infection [21,23,27]. Within the population that was under study, the causal pathogens staphylococcus and streptococcus species had the highest prevalence [20,24,25,26,28]. *S. aureus* and coagulase-negative staphylococci were the types of staphylococci that were discovered the most frequently [19,20,21,22,24,26,28], as presented in Figure 2, where 33.4% of infections were caused by S. aureus, and32.0% were caused by species of streptococci, respectively. *S. bovis* and *viridans* streptococci were the types of streptococci that were found the most frequently [21,22]. Enterococci, Gram-negative rods, and fungal pathogens were among the other categories of infectious agents that were analyzed. Although included articles were quite heterogeneous regarding the prevalence of different types of staphylococci and streptococci, studies that took place in recent years have found a higher prevalence of staphylococci [19,20,28]. However, some report a similar prevalence between the two species [25,27].

Figure 2 shows the mean prevalence of each type of pathogen for the included studies. Regarding comorbidities, each study included a substantial number of participants with various comorbidities. Conditions related to the heart and diabetes were the most common, followed by kidney disease, lung disease, cancer, and hepatic disease. Regarding unfavorable outcomes, the mortality rate ranged from 16% to 42.1% on average [19,20,21,22,23,24,25,26,27,28], and it was consistently high across all of the studies. Clinical information and outcomes are presented in Table 2. Complications such as acute heart failure, acute kidney injury, and sepsis were observed in patients who were diagnosed with IE [19,20,22,23]. As of diagnosis, the majority of studies relied on transthoracic echocardiography (TTE) or transesophageal echography (TOE), in addition to blood cultures [20,21,23,25,26,27,28]. During the course of the investigation, patients diagnosed with IE were evaluated using a modified version of the Duke criteria [22,23,25,26,27,28]. The major criteria included the detection of two positive blood cultures with organisms consistent with IE and confirmation of endocardial involvement identified by echocardiography. Other minor criteria, such as comorbidities or other risk factors, were also included in the list [28]. Patients described in these studies presented with a series of symptoms such as fever [22,23,25,28], heart murmur, anemia, or other systemic symptoms such as weight loss or anorexia [25,28]. 

Cardiac complications, sepsis, renal problems, neurological problems, an increase in age, and prosthetic endocarditis were the most frequent risk factors associated with an increased mortality rate. Table 3 displays the most common risk factors discovered in the studies that were included in the review, together with the statistical results that correspond to those risk factors as an outcome of the multivariate analyses of predictors that were carried out. As seen in Table 3, the variables that had the highest odds ratio of occurring were septic shock, with an OR of 20.26 (4.04—1–2.74) in one study [25] and 12 (6–24) in another [27], and, respectively, heart failure, OR = 4.80 (3.59–71.53) [27] and renal failure, OR = 4.8 (1.8–13.1) [23] and HR = 8.68 (2.32–32.4) [28]. In addition to the most common causes, certain studies discovered that metabolic illnesses, vascular problems [24], antibiotic resistance [19], and the absence of surgery [21] were statistically significant as death risk factors.

Among protective factors identified in the included studies, it has been discovered that early consultation and surgery manage to adjust the death rate at a reduced incidence [20,25,27,28]. Every research study found that the elderly had a greater likelihood of experiencing poor outcomes compared to the younger group [19,21,22,23,26,27,28]. Native IE was discovered more frequently than prosthetic IE [22,24,25,28]. In the investigations that involved surgical intervention [19,27], the types of surgery that were carried out were the replacement of the aortic valve, replacement of the mitral valve, replacement of both valves, and repair of the mitral valve. In terms of other types of treatments, such as the use of antibiotics, it has been reported that surgery remains the best treatment option [21]. However, in cases in which the patient is at high risk of mortality because of surgical intervention, medical treatment will be preferred [28].

## 4. Discussion

The current study described how infective endocarditis exhibits a nonspecific clinical presentation but has a significant mortality and morbidity rate among the elderly individuals [29]. This comprehensive analysis aimed to investigate the clinical manifestations of infective endocarditis in the older population, with a particular focus on the risk factors that indicate a greater mortality rate. It has been discovered that the majority of the causal pathogens are staphylococci and streptococci [20,24,25,26,28], with staphylococci having a frequency that is slightly higher than that of streptococci (34.0% vs. 32.0%), among 2222 patients that were analyzed in 10 studies. This result upholds the conclusions derived from other studies, which either reported an increased incidence of staphylococci species [30] or an equal proportion of both types of pathogens [17,31]. However, in line with the results of other studies, most of the species detected were *S. aureus, S. bovis,* and other strains of coagulase-negative staphylococci and *viridans* streptococci [2,17,31]. 

The strains of the relevant microorganisms that have developed resistance to antibiotics are the source of the problem that has arisen. For instance, the staphylococcus species have a greater possibility of being antibiotic resistant, which is the reason why variants that are resistant to methicillin have arisen [8,24,32]. In the same manner, the rates of staphylococcal pathogens remain high because of their high susceptibility in the healthcare sector, following different invasive procedures [8]. Because of this, there is a concern regarding the transmission of these infections, in conjunction with the hazards that the more sensitive population confronts, highlight the need to create better strategies and guidelines in the management of IE in elderly patients [32]. Another illustration of this is the rise in the number of cases of enterococci among the elderly. Types of enterococci, such as *E. faecalis* and *E. faecium,* occur more in the elderly and, respectively, are resistant to vancomycin, aminoglycosides, and ampicillin [8]. In addition to that, the use of medication that is required in the treatment of different comorbidities that the older age group experiences consist of the nature of the medication that they are taking. More precisely, different types of diseases require medication that could lead to different side effects that will raise the risks to which they are exposed [19]. Nephrotoxicity [19] and cardiac arrhythmias such as atrial fibrillation [28] are two of the potential adverse effects. Moreover, except for one study, all remaining studies found a higher prevalence of males with infective endocarditis [19,20,21,22,24,25,26,27,28]. The higher prevalence of male patients has been described in other studies, although an identifiable factor responsible for this particularity [2,8,17]. 

When it comes to diagnosis, studies included relied on echocardiography and blood cultures. According to the Duke criteria used in the following clinical criteria, transesophageal echocardiography and transthoracic echocardiography are recommended for prosthetic valves and for native valves, respectively [8]. The elderly had a larger risk of developing an intracardiac device infection as compared to the younger population [22], with the mitral valve being the most common valve affected [19,20,22,23,26]. However, one study found that when the cohort was narrowed down to non-drug users, there were no differences between the younger population and the older age population. These results are in agreement with other articles which found that IE occurs more frequently on the mitral valve in the elderly population [3,5]. There were no significant differences across the cohorts with regard to the symptoms that the patients had described, such as fever or arrhythmia [23,28]. Concerning the prevalence of comorbidities, the majority of research [19,23,24,25,26,27,28] found that cardiac problems, diabetes, and renal disorders were more common. This result is in line with the findings of other studies [3,33] that discovered a connection between the aforementioned diseases and IE. Comorbidities can become risk factors because of the problems that they may include [16], which can lead to greater rates of adverse outcomes and difficulties in following a treatment for IE that would not interfere with the pre-existing disease [34].

When taking into consideration the mortality rate, the findings were consistent with those of earlier studies, which demonstrated an increasing mortality rate [1,3,4]. Because of this unfavorable outcome, there is an increased demand for the discovery and implementation of efficient population-specific methods by which IE could be properly treated [30,33]. The management of IE should begin as soon as possible, according to existing studiers and guidelines [3,10], which state that due to the atypical presentation that IE has and the major complications that can occur, such as sepsis or septic shock, IE should be treated as quickly as possible in order to reduce the risk of developing a more severe form of IE. Even though early surgery appears to be a protective factor against adverse outcomes [20,25,27,28], there are still important limitations in providing this type of treatment for elderly patients. The most important of these limitations in considering this treatment is clinical complications that could increase the risk of mortality, along with increased age [3]. Surgery is not typically recommended for patients older than 80 years old, due to the increased likelihood of death following the procedure, which outweighs its benefits [15]. This review discovered a higher frequency of community-acquired IE [20,24,25,26,28], despite the fact that certain research discovered that IE in the elderly is more likely to be obtained in a hospital setting [16,31].

Cardiac comorbidities, sepsis, advanced age, renal disease, neurological problems, and being diagnosed with prosthetic infective endocarditis were the most common risk factors that led to an elevated mortality rate [19,20,21,22,23,24,25,26,27,28]. This may happen as a consequence of the fact that seniors have a greater likelihood of experiencing major complications. This is due to the fact that many elderly people have underlying conditions that raise the risk of developing major incidences. Furthermore, elderly people are more likely to contract pathogens because of healthcare-associated procedures, such as hemodialysis or cardiac implantable electronic devices [3]. As a consequence of this, the decreased utilization of surgical procedures is an additional element that contributes to the upkeep of a higher death rate [35]. We were able to identify significant variations between the results of IE when we considered the fact that the majority of the studies included made comparisons between the elderly population and the younger population. Nevertheless, the elderly population represents a vulnerable age group since they have a higher probability of dying due to a higher prevalence of comorbidities and higher incidence of complications [19,20,21,22,23,26,27,28]. 

This current systematic review has several limitations. First, the number of studies that were assessed was rather limited, consisting only of ten studies. Furthermore, the sample size of the cohort that was examined in the studies was also extremely limited, with most of the studies having up to 200 patients each [19,20,21,23,24,25,27,28]. Since only seven countries were examined in this study, it is not possible to apply these findings to the entire world. At the same level, although all studies implied an older age population, some studies had a range of years closer to 60 years old [19,20,25], while others focused more on the population above 70 [21,27]. At the same level, studies used different methods of categorizing pathogens, with some of them only recording the major classifications (streptococci, staphylococci, enterococci) [23,25,27]; therefore, information about the most prevalent pathogen could not be extracted. In the same manner, there was a dispersed way of identifying the pathogens [19,20,21,22,23,24,25,26,27,28]; thus, future studies could concentrate on using the same pathogens to draw an accurate conclusion on the ones that are the most prevalent and contain the highest risks. 

It is possible for future research to make use of age stratification, taking into consideration the fact that recent studies have shown that, in addition to the difference that exists between populations of young people and populations of older people, there are also differences that exist within groups. These intragroup differences are most prevalent among the elderly, since the chances of treatment success significantly decrease once a person reaches the age of 80 or older [15]. In addition, each study had a unique perspective on the characteristics that represented a risk, which made it challenging to classify predictors in an effective manner due to the fact that they were relatively heterogeneous. In subsequent research, the focus may be placed on particular classes of risk factors, with the goals of identifying those that are most significant and locating effective treatment strategies for the condition. To this end, there is a pressing need for more research to be conducted that focuses on the treatment management of older patients, particularly randomized controlled trials, which continue to be the gold standard for evaluating the efficacy of various treatment modalities [3]. Since IE continues to present a significant obstacle for experts [10], there is an urgent need to conduct more recent research on the various methods of treatment. This is especially important when considering the fact that the majority of the published research on the elderly population consists of articles published more than ten years ago [22,23,26,27]. Nonetheless, better outcomes could be increased if there is a multidisciplinary approach, coming from physicians, geriatricians, cardiologists, cardiac surgeons, and microbiologists altogether, that could assess infective endocarditis globally and, thus, find the best therapeutic approaches for this specific age group [3,33,36]. 

## 5. Conclusions

Elderly patients seem to be the most affected by IE, as older age is a significant risk factor that contribute to negative outcomes, among other complications that they encounter. These factors make IE a condition that demands immediate medical attention. Even though IE has been researched for a considerable amount of time, the incidence of mortality is still very high, particularly among the more susceptible population, which includes those of older age. Taking into consideration the fact that most of the elderly patients with IE suffer from significant complications and are unable to undergo surgery due to an increased risk of complications after the procedure, it is imperative that effective treatment methods be investigated.

## Figures and Tables

**Table 1:** Demographic characteristics of the included studies.

Study	Country	Study Cohort	Age (Mean)	Gender (Males)
Budea et al. (2022) [19]	Romania	131	67.5	51.9%
Chirio et al. (2018) [20]	France	100	68	69.0%
Di Salvo et al. (2013) [21]	France	87	77	58.0%
Durante-Mangoni et al. (2008) [22]	USA	1056	>65 (NR)	64.2%
Gagliardi et al. (1998) [23]	USA	44	72	ND
Hase et al. (2015) [24]	Japan	180	69	60.6%
Marques et al. (2019) [25]	Portugal	134	>75 (NR)	73.1%
Ramirez-Duque et al. (2011) [26]	Spain	356	>64 (NR)	63.3%
Remadi et al. (2009) [27]	France	75	79.8	62.6%
Wu et al. (2019) [28]	China	59	72	69.5%

ND (no data); NR (not reported).

**Table 2:** Clinical characteristics of included studies and negative outcomes.

Study	Causative Pathogens	Comorbidities	Type of Infection		Mortality Rate
			Community-Acquired IE	Healthcare-Associated IE	
[19]	*Staphylococcus aureus* 22 9.0% Coagulase-negative staphylococci 14.5% Streptococcus spp. 15.3% *Enterococcus faecalis* 13.0% Gram-negative rods 13.8% Fungi 14.5%	Cardiac 46.6% Metabolic 22.1% Cerebrovascular 36.6% Digestive and liver 26% Kidney disease 10.7% Cancer 13.7%	-	100%	40.5%
[20]	*Staphylococcus aureus* 29% Oral streptococci 25% *Enterococcus spp.* 12% Coagulase-negative staphylococci 10% *Streptococcus gallolyticus* 10% Fungi 2%	Congestive heart failure 19% Diabetes mellitus 18% Immunodeficiency 14% Chronic kidney impairment 11% Liver disease 11% Pulmonary disease 7%	73%	27%	22%
[21]	*Staphylococcus aureus* 17% *Streptococcus Bovis* 16% Enterococci 9% Fungi 1%	Heart disease 49%	NR		17%
[22]	*Staphylococcus aureus* 27.8% Coagulase-negative staphylococci 11% Enterococci 17.6% *Streptococcus Bovis* 8.9% Viridans streptococci 13.1% Fungi 1.5%	Diabetes 21% Cancer 3%	28.20%	71.8%	25.8
[23]	Streptococcus species 43% Staphylococcus species 36% Gram-negative rods 9%	Diabetes 14% Heart disease 30% Renal disease 14% Hypertension 45% Cancer 20%	NR	NR	27%
[24]	*Staphylococcus aureus* 27.2% Viridans streptococci 22.2% Coagulase-negative staphylococci 11.7%	Diabetes 13.8% Cancer 8.3%	58.9%	15%	26.1%
[25]	Streptococcus species 31.3% Staphylococcus species 30.6% Enterococci species 11.9 Gram-negative rods 3.7 Fungi 2.2%	Hypertension 50.7% Heart disease 49.3% Hepatic disease 23.9% Diabetes 15.8% Pulmonary disease 15.7% Renal disease 11.9%	77.6%	22.4%	31.3%
[26]	Viridans streptococci 16.9% *Staphylococcus aureus* 17.4% Coagulase-negative staphylococci 17.1% Enterococci 16.3% Gram-negative rods 4.2% Fungi 1.1%	Diabetes Renal disease Hepatic disease Cancer Pulmonary disease	78.9%	21.1%	42.1%
[27]	Streptococcus species 37.4% Staphylococcus species 36%	Diabetes 25.3% Renal failure 17.3% Cancer 26.7%	NR	NR	16%
[28]	Viridans streptococci 5.1% *S. aureus* 3.4% Coagulase-negative staphylococci 15.3% Enterococci 1.7% Fungi 1.7	Heart disease 18.6% Hypertension 42.4% Diabetes 16.9	79.7%	10.2%	20.3%

**Table 3:** Predictors of mortality in IE.

Mortality Risk Factors	Study	OR	95% CI	*p*-Value
Cardiac disease	1 [19]	4.07	3.44–6.90	<0.001
	6 [24]	3.50	1.53–8.00	0.001
	7 [25]	4.80	3.59–71.53	0.003
	8 [26]	3.47	2.42–4.98	<0.01
Septic shock	1 [19]	6.19	4.15–8.28	<0.001
	7 [25]	20.26	4.04–102.74	<0.001
	8 [26]	3.98	2.50–6.31	<0.01
	9 [27]	12.00	6.00–24.00	0.001
Renal disease	1 [19]	3.14	2.36–4.03	0.001
	5 [23]	4.80	1.80–13.10	0.00
	8 [26]	1.73	1.23–2.43	<0.01
	10 [28]	8.68	2.32–32.40	0.001
Old age	2 [20]	11.2	2.76–46.17	<0.001
	3 [21]	3.93	1.59–9.73	0.003
	4 [22]	2.04	1.62–2.56	<0.001
	8 [26]	1.02	1.01–1.03	<0.01
Neurological problems	3 [21]	4.25	1.50–12.30	0.006
	5 [23]	4.00	1.30–12.20	0.02
	8 [26]	1.96	1.38–2.79	0.01
	9 [27]	3.00	1.10–7.50	0.02
Prosthetic valve endocarditis	3 [21]	4.73	1.76–12.69	0.002
	8 [26]	2.11	1.47–3.03	<0.01

OR (odds ratio), HR (hazard ratio), 95% CI (confidence interval).

## Data Availability

Not applicable.
